# Effect of statins on aortic root growth rate in patients with bicuspid aortic valve anatomy

**DOI:** 10.1007/s10554-015-0749-0

**Published:** 2015-08-29

**Authors:** Madelien V. Regeer, Philippe J. van Rosendael, Vasileios Kamperidis, Martin J. Schalij, Jeroen J. Bax, Nina Ajmone Marsan, Victoria Delgado

**Affiliations:** Heart Lung Center Leiden, Leiden University Medical Center, Leiden, The Netherlands; Department of Cardiology, Leiden University Medical Center, Albinusdreef 2, 2333 ZA Leiden, The Netherlands

**Keywords:** Bicuspid aortic valve, Aortic dilation, Echocardiography, 3-Hydroxy-3-methylglutaryl coenzyme A reductase inhibitors

## Abstract

Bicuspid aortic valve (BAV) anatomy is associated with increased growth rate of the aortic root compared to tricuspid aortic valves. Statins decrease the growth rate of abdominal aneurysms; however their effect on the aortic root growth rate has not been elucidated. The present study evaluated the association between use of statins and aortic root growth in patients with BAV. A total of 199 patients (43 ± 15 years, 69 % male) with BAV who underwent ≥2 echocardiographic measurements of the aortic root ≥1 year apart were included in this retrospective observational study. Median follow-up duration was 4.7 years (interquartile range 2.7–8.3 years). Growth rate (mm/year) of the aortic root was compared between statin users (n = 41) and non-users (n = 158). Statin users were significantly older and had more cardiovascular risk factors than their counterparts. Ascending aorta diameter was significantly smaller at baseline and at follow-up in statin users compared with non-users when adjusted for coronary artery disease, age and medication. The average annual growth rate was 0.08 mm/year (95 % confidence interval 0.03–0.13) for the aortoventricular junction, 0.16 mm/year (0.11–0.21) for the sinus of Valsalva, 0.12 mm/year (0.07–0.17) for the sinotubular junction and 0.45 mm/year (0.37–0.53) for the ascending aorta. The dilation rate of the aortic segments was not different between statin users and non-users. In conclusion, in patients with BAV, although the use of statins was associated with smaller ascending aorta, the annual dilation rate of the aortic root was not influenced by the use of statins.

## Introduction

Patients with bicuspid aortic valve (BAV) anatomy have shown larger aortic root diameters and faster growth rate of the ascending aorta compared with patients with tricuspid aortic valve (TAV) anatomy leading to an excess risk of dissection and rupture in the former patients [1]. The average growth rate of the ascending aorta in patients with BAV is 0.77 mm/year, fivefold higher than that of age-matched TAV patients [2]. Pathophysiological factors associated with the increased prevalence of ascending aorta dilation and faster growth rate among BAV patients include abnormal wall stress distribution (particularly in patients with dysfunctional aortic valves), altered aortic wall structure and/or an underlying genetic substrate [3]. Specifically, increased activity and expression of metalloproteinases have been demonstrated in histological samples of ascending aorta aneurysms of patients with BAV [4]. The pleiotropic effects of 3-hydroxy-3-methylglutaryl coenzyme A reductase inhibitors (statins) reduce the expression of metalloproteinases [5, 6] and have been demonstrated to limit the growth rate of abdominal aneurysms and to improve the thoracic and abdominal aortic aneurysm rupture and dissection-free survival [7–11]. In patients with BAV, the use of statins has been associated with smaller aortic root and ascending aorta diameters in cross-sectional studies [12]. However, the effect of statins on the aortic root and ascending aorta growth rate of these patients remains unclear. Accordingly, we evaluated the effect of statins on the aortic root and ascending aorta growth rates of patients with BAV in a retrospective study.

## Methods

### Patients

**Fig. 1:**
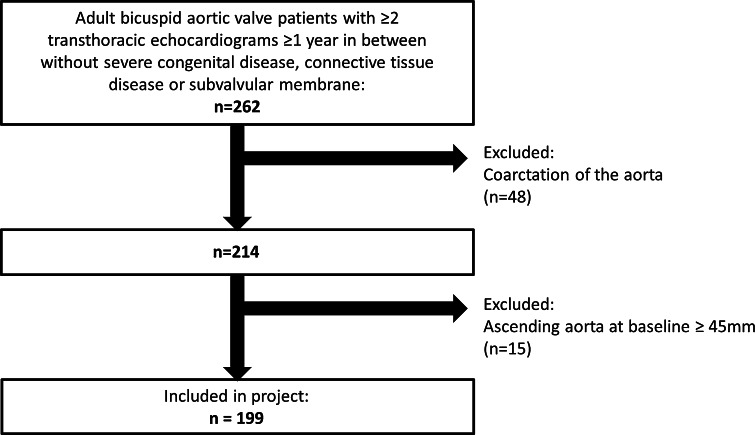
Flowchart of patient inclusion

The present retrospective study included patients aged ≥18 years with BAV who underwent transthoracic echocardiography surveillance from 1995 to 2014 for at least 1 year. If patients underwent aortic valve and/or aortic root surgery during follow-up, the last transthoracic echocardiography before surgery was selected for analysis. Patients with complex congenital heart disease, connective tissue disease and subvalvular aortic stenosis were not included. In total 262 patients were eligible for inclusion. From this group, 48 patients with coarctation of the aorta and 15 patients with an ascending aorta of ≥45 mm at baseline were excluded. The final cohort consisted of 199 patients (Fig. 1).

Demographic and clinical characteristics were collected in the departmental Cardiology Information System (EPD-Vision^®^, Leiden University Medical Center, Leiden, The Netherlands) and retrospectively analyzed. Medication use (statins, angiotensin converting enzyme-inhibitors/angiotensin receptor blockers and beta-blockers) were obtained from chart review. Patients were divided into two groups: patients treated with statins for at least 50 % of the follow-up time (statin users) and patients not treated with statins (non-users). Aortic root diameters were measured at four levels and annual growth rate in mm/year was compared between the two groups. The institutional review board approved this retrospective analysis of clinically acquired data and waived the need for patient written informed consent.

### Two-dimensional transthoracic echocardiography

Transthoracic echocardiography was performed using commercially available ultrasound systems (System Five, Vivid 7, and E9, General Electric Healthcare, Vingmed, Horten, Norway) equipped with 3.5-MHz or M5S transducers. Parasternal and apical views were obtained at rest with patients in the left decubitus position while subcostal and supra-sternal views were obtained with patients in supine position. Two-dimensional, M-mode and Doppler data were acquired according to current recommendations [13]. The echocardiographic data were digitally stored in cine-loop format and data were retrospectively analysed off-line using commercially available software (EchoPac 112.0.1, GE Medical Systems, Horten, Norway). Left ventricular (LV) end-diastolic and end-systolic diameters were measured in the M-mode parasternal long-axis view recordings. LV end-diastolic and end-systolic volumes were measured in the apical 2- and 4-chamber views and LV ejection fraction was calculated according to the Simpson’s biplane method [13].

BAV was diagnosed on the parasternal short-axis view of the aortic valve by the presence of two commissures in systole [14]. BAV was typical when the commissures were oriented at 4–10, 5–11 or 3–9 o’clock and atypical when the commissures were oriented at 1–7 or 12–6 o’clock. The presence of a raphe was noted [14]. Aortic valve function was evaluated using colour-Doppler, continuous- and pulsed-wave Doppler. Valvular dysfunction was defined as pure aortic regurgitation when ≥grade 2 and less then mild aortic stenosis, pure aortic stenosis when ≥moderate and aortic regurgitation ≤grade 1 or mixed aortic valve disease [15, 16].

Aortic root dimensions were measured using the leading edge-to-leading edge technique during end-diastole in the parasternal long-axis view perpendicular to the long-axis of the aorta at four predefined levels: (1) the aortoventricular junction (AVJ) defined as the hinge points of the aortic leaflets, (2) the sinuses of Valsalva (SOV), (3) the sinotubular junction (STJ) and (4) the ascending aorta (AAo) (4 cm distal from the aortic valve) [17, 18].

### Statistical analysis

Continuous variables were reported as mean ± standard deviation or median and interquartile range when appropriate. Categorical variables were reported as numbers and percentages. Continuous and categorical variables were compared with the Student’s *t* test (or Mann–Whitney *U* test in non-normally distributed variables) and Chi square test, respectively. Differences in aortic root diameters at baseline and follow-up between groups were assessed with ANOVA test for repeated measures. Statin use was incorporated in the model as factor together with coronary artery disease, use of beta-blockers and the use of angiotensin converting enzyme-inhibitors or angiotensin receptor blockers. Age at baseline was incorporated in the model as covariate. Estimated marginal mean ± standard error of the mean for the aortic root diameters were reported for statin users and non-users at baseline and during follow-up. Regression analysis was used to assess the difference in aortic root growth rate in mm/year between statin users and non-users following the previously described instrumental variables approach [19]. Assuming that the aortic root growth is linear, the estimate of the association between aortic root growth and the follow-up was obtained by linear regression analysis without including an intercept. Next statin use multiplied by follow-up duration in years was introduced to assess the additive value of statin use on the growth of the aortic root in mm/year. Intra-observer variability was assessed using Bland–Altman method measuring 20 echocardiograms repeatedly by one observer. Similarly, inter-observer variability was assessed by performing the measurements by two independent observers at least 1 week apart. All statistical tests were two-sided. A *p* value <0.05 was considered statistically significant. Data analyses were performed using the SPSS software (Version 20.0. Armonk, NY: IBM Corp).

## Results

A total of 199 patients (mean age 43 ± 15 years, 69 % men) were included in the present analysis. There were 41 (21 %) statin users and 158 (79 %) non-users. Baseline characteristics are presented in Table 1. Among statin users, the median duration of the treatment was 3.5 years (interquartile range 2.4–5.4 years). The types and doses of statins varied over time and were adjusted to patient’s tolerance. The majority of patients received simvastatin (usually 20 or 40 mg daily) or rosuvastatin (usually 10 mg daily). Other types of statins used were atorvastatin and pravastatin (usually 20 or 40 mg daily). Statin users were significantly older, with greater incidence of coronary artery disease, hypertension and New York Heart Association II–III functional class heart failure symptoms. Furthermore, statin users received more frequently angiotensin converting enzyme-inhibitors/angiotensin receptor blockers and beta-blockers. There was no significant difference between groups in total cholesterol levels at baseline. However, in statin users there was a tendency towards higher LDL-cholesterol and higher triglycerides. Furthermore the HDL-cholesterol was significantly lower in statin users compared to their counterparts. There was no significant difference in LV end-diastolic and end-systolic dimensions. Statin users had slightly lower LV ejection fraction compared to non-users (51 ± 7 vs. 53 ± 7 %; *p* = 0.054). The presence of a raphe, type of BAV and aortic valvular dysfunction was not significantly different between statin-users and non-users.

**Table 1:** Baseline characteristics

Variable	Non-users (n = 158)	Statin users (n = 41)	*p* value
Age (years)	40 ± 14	55 ± 10	<0.001
Male	106 (67 %)	31 (76 %)	0.389
Body surface area (m^2^)	1.93 ± 0.23	1.98 ± 0.21	0.189
Smoking	29 (18 %)	12 (29 %)	0.186
Diabetes	4 (3 %)	3 (7 %)	0.314
Hypertension	26 (16 %)	14 (34 %)	0.021
NYHA functional class			<0.001
I	145 (91 %)	27 (66 %)	
II	9 (6 %)	12 (29 %)	
III	4 (3 %)	2 (5 %)	
IV	0 (0 %)	0 (0 %)	
Previous cardiac surgery	4 (3 %)	4 (10 %)	0.098
Coronary artery disease	1 (1 %)	15 (37 %)	<0.001
ACE-inhibitor or ARB	32 (20 %)	25 (61 %)	<0.001
Beta-blocker	32 (20 %)	24 (59 %)	<0.001
Total cholesterol (mg/dl)	195 ± 36	198 ± 47	0.690
LDL cholesterol (mg/dl)	120 ± 32	136 ± 46	0.071
Triglycerides (mg/dl)	128 ± 63	154 ± 85	0.081
HDL cholesterol (mg/dl)	57 ± 17	49 ± 14	0.028
LV end-diastolic diameter (mm)	50 ± 6	49 ± 6	0.160
LV end-systolic diameter (mm)	31 ± 6	31 ± 7	0.994
LV end-diastolic volume (ml)	131 ± 39	120 ± 27	0.089
LV end-systolic volume (ml)	62 ± 24	59 ± 17	0.450
LV ejection fraction (%)	53 ± 7	51 ± 7	0.054
Atypical bicuspid aortic valve	45 (28 %)	11 (27 %)	0.988
Raphe	135 (85 %)	37 (90 %)	0.586
Valvular dysfunction			0.274
None	99 (63 %)	27 (66 %)	
Pure aortic regurgitation	27 (17 %)	6 (15 %)	
Pure aortic stenosis	21 (13 %)	8 (19 %)	
Mixed aortic disease	11 (7 %)	0 (0 %)	

Data are presented as mean ± SD or as number (percentage)

*ACE* angiotensin converting enzyme, *ARB* angiotensin receptor blocker, *HDL* high density lipoprotein, *LDL* low density lipoprotein, *LV* left ventricular, *NYHA* New York Heart Association

**Fig. 2:**
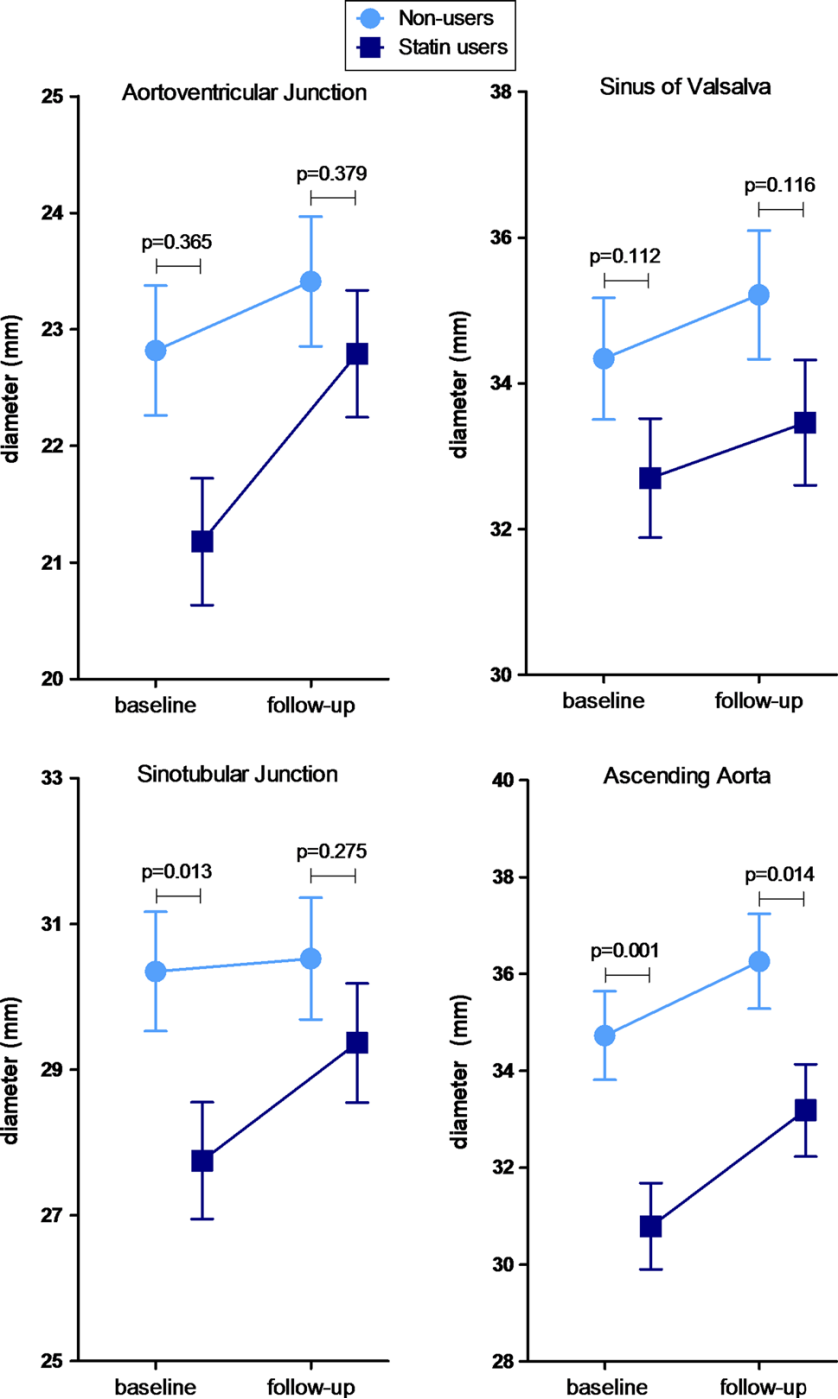
Comparison of aortic diameters at baseline and at follow-up between statin users and non-users. Data are presented as estimated marginal mean ± standard error of the mean calculated for mean age at baseline of 43 years and corrected for the presence of coronary artery disease and the use of angiotensin converting enzyme-inhibitors or angiotensin receptor blockers and beta-blockers

Echocardiographic follow-up was complete with a median follow-up duration of 5.1 years (interquartile range 2.7–8.8 years) in non-users and 3.9 years (interquartile range 2.6–5.5 years) in statin users (*p* = 0.052). Figure 2 shows the differences in aortic root diameters at baseline and during follow-up between the groups, using the ANOVA test for repeated measurements adjusted for age at baseline, the presence of coronary artery disease and the use of angiotensin converting enzyme-inhibitors or angiotensin receptor blockers and beta-blockers. The aortic root diameter at the level of the AVJ was not significantly different between statin users and non-users. Aortic root diameter at the level of the SOV was comparable at baseline and at follow-up in statin users (32.7 ± 0.8 and 33.5 ± 0.9 mm) compared to non-users (34.3 ± 0.8 and 35.2 ± 0.9 mm). At the level of the STJ, the diameter was significantly smaller in statin users compared to non-users at baseline (27.8 ± 0.8 vs. 30.3 ± 0.8 mm; *p* = 0.013). However, there was no significant difference in STJ diameter at follow-up. The AAo was significantly smaller in statin users compared to non-users at baseline (30.8 ± 0.9 vs. 34.7 ± 0.9 mm; *p* = 0.001) and at follow-up (33.2 ± 1.0 vs. 36.3 ± 1.0 mm; *p* = 0.014).

The average annual aortic growth rate was 0.08 mm/year (95 % confidence interval 0.03–0.13) for the AVJ, 0.16 mm/year (0.11–0.21) for the SOV, 0.12 mm/year (0.07–0.17) for the STJ and 0.45 mm/year (0.37–0.53) for the AAo. There was no significant additive value of statin use on the annual growth rate of the aortic segments as presented in Table 2.

**Table 2:** Average annual growth rates in mm/year per aortic segment with the additive effect of the use of statins on the annual growth rate

Variable	Annual growth rate		Additive effect of the use of statins on annual growth rate	
	B (95 % CI)	*p* value	B (95 % CI)	*p* value
Aortoventricular junction	0.08 (0.03–0.13)	0.001	−0.03 (−0.16 to 0.11)	0.687
Sinus of Valsalva	0.16 (0.11–0.21)	<0.001	−0.06 (−0.20 to 0.08)	0.413
Sinotubular junction	0.12 (0.07–0.17)	<0.001	0.13 (−0.02 to 0.28)	0.079
Ascending aorta	0.46 (0.37–0.54)	<0.001	−0.05 (−0.27 to 0.17)	0.671

Data are presented as regression coefficient (B) and 95 % confidence interval (95 % CI) indicating annual growth rates in mm/year

The intra-observer variability, displayed as 1.96 times the standard deviation of the difference between the two measurements was 1.6 mm for the AVJ, 3.2 mm for the SOV, 4.2 mm for the STJ and 5.1 mm for the AAo. The inter-observer variability was 2.6 mm for the AVJ, 3.9 mm for the SOV, 4.3 mm for the STJ and 4.4 mm for the AAo.

## Discussion

The main results of the present observational study can be summarized as follows: among patients with BAV, those treated with statins had smaller STJ at baseline and smaller AAo at baseline and at follow-up compared with patients non-treated with statins after correcting for age, presence of coronary artery disease and the use of angiotensin converting enzyme-inhibitors or angiotensin receptor blockers and beta-blockers. However, there was no significant effect of statins on the annual aortic growth rate since this was comparable between both subgroups of patients.

Bicuspid aortic valve is the most frequent congenital heart disease with an estimated prevalence of 1.3 % in the general population [20]. In addition, BAV anatomy is associated with aortopathy and increased prevalence of aneurysms of the ascending aorta which occur at a younger age than in TAV [2, 21]. Specific genotypes, histological and inmunohistochemical factors and changes in hemodynamics with increased shear stress on the aortic wall have been associated with increased risk of ascending aortic dilation in patients with BAV [21–23]. Indeed, in patients with BAV the growth rate of the aortic root and ascending aorta ranges between 0.2 and 0.9 mm/year [9, 24], a growth rate relatively similar to that observed in patients with Marfan syndrome but significantly different from that of patients with TAV and degenerative aortopathy [24]. In a recent study involving 353 patients with BAV who were compared with 51 patients with degenerative aortopathy and 50 patients with Marfan syndrome, Detaint et al. [24] showed that in patients with BAV the maximal dilation rate was 0.42 ± 0.6 mm/year while in patients with Marfan syndrome and in patients with degenerative aortopathy the maximal dilation rate was 0.49 ± 0.5 and 0.20 ± 0.3 mm/year, respectively (*p* = 0.02). Interestingly, the growth rate was largest at the ascending aorta in patients with BAV (0.42 ± 0.6 mm/year) while in patients with Marfan syndrome, the sinuses of Valsalva showed the largest growth rate (0.49 ± 0.5 mm/year). The present study shows similar growth rates for each level of the aortic root and ascending aorta to those reported by Detaint et al. with the ascending aorta as the segment with the largest growth rate. However, Detaint et al. [24] did not find an independent association between aortic growth rate and the use of angiotensin converting enzyme inhibitors, angiotensin receptor blockers or beta-blockers. The present study also did not find an association between the use of angiotensin converting enzyme-inhibitors/angiotensin receptor blockers and beta-blockers and the aortic root diameters at baseline and during follow-up. In contrast, the present study provides information on the association between statin use and the aortic growth rate of these patients.

The pathogenesis of ascending aorta dilation in patients with BAV is multifactorial and, in contrast to abdominal aortic aneurysms, ascending aorta aneurysms do not result in atherosclerosis [25]. However, in patients with BAV, there is an increased expression and activity of metalloproteinases that degrade the type IV collagen, elastin and fibrillar collagens [4]. Statins, clinically used as lipid lowering drugs, have various anti-inflammatory and pleiotropic effects [6]. Therefore it has been hypothesized that statins may have an inhibitory effect on aneurysm formation. For example, in experimental models statins have shown to inhibit the progression of abdominal aortic aneurysms independently of the total serum cholesterol levels lowering effect [5, 6]. Inhibition of macrophage infiltration into the aorta and preservation of aortic wall elastin and medial smooth muscle cells in the aortic wall are the main underlying mechanisms explaining those findings [5, 6].

Clinical experiences have shown that patients with BAV who receive statin therapy have smaller ascending aorta diameters compared to non-users [12]. In 147 patients with BAV undergoing aortic valve replacement with or without aortic root replacement, Goel et al. showed that patients treated with statins had significantly smaller ascending aorta diameters than patients not receiving statins (3.6 ± 0.7 vs. 3.9 ± 0.6 cm, *p* < 0.001) and the use of statins was independently associated with a 0.33 cm reduction in aortic size [12]. Furthermore, Jovin et al. [9] showed that in patients with thoracic aneurysms (without specifying the prevalence of BAV), the annual growth rate of the thoracic aortic aneurysm in patients treated with statins (n = 46) was comparable to that of patients not treated with statins (n = 169) (0.8 vs. 0.9 mm/year, *p* = 0.7). Angeloni et al. [11] recently published a large propensity score matched cohort study that showed a significantly smaller ascending aorta dilation rate of 0.95 mm/year in statin users compared to 1.27 mm/year in non-users. The reported dilation rates were both larger than those reported in the present study, which might be explained by the inclusion of larger ascending aortic aneurysms at baseline in the study by Angeloni et al. [11]. However, it should be emphasized that the anatomy of the aortic valve was not specified and that the age of the population was older than that of the patients included in our study and therefore, the results reported by Angeloni and coworkers may not be extrapolated to a younger BAV patient cohort.

### Clinical implications

Ascending aorta dilation is the second most common morbidity in patients with BAV [1]. Due to the associated risk of aneurysm rupture, identification of pathophysiological determinants and potential therapeutic targets to reduce the risk of aortic dilation is a cardiovascular research priority. Besides advances in multimodality imaging that permit accurate diagnosis and characterization of BAV and its hemodynamics; genetic- and biomarker-based risk stratification may help to identify those patients at increased risk of aortic dilation. In addition, prospective studies evaluating the effect of specific therapies such as statins that may reduce the expression and activity of metalloproteinases or angiotensin converting enzymes inhibitors that may favourably affect the shear strain of the aortic wall are needed.

### Limitations

The present study, taking into consideration all the limitations of an observational retrospective design, shows that the use of statins is associated with smaller aortic root and ascending aorta dimensions in patients with BAV but does not influence the growth rate. Therefore, it cannot be concluded that BAV patients without an indication for statins, should use a statin as primary prevention for aortic dilation. The relatively small number of patients treated with statins is also another limitation. In addition, patients using statins were different from non-users regarding age, presence of coronary artery disease and the use of angiotensin converting enzyme-inhibitors, angiotensin receptor blockers and beta-blockers. We corrected the statistical analysis for these important confounding factors. However, the influence of potential other unknown confounding factors could not be taken into account. Prospective trials with appropriate sample sizes randomizing patients to statin treatment or placebo are needed to assess the isolated effect of statins on aortic root dilation excluding potential confounding factors (such as cardiovascular risk factors and other drugs used). Other limitations of the present study include the evaluation of a selected cohort of patients with at least two echocardiographic studies ≥1 year of follow-up. This may have introduced selection bias, since patients with large or rapid growing aneurysms will be referred immediately for surgery. Furthermore, compared with other imaging techniques that provide higher spatial resolution data, transthoracic echocardiography may provide less accurate measurements of the aortic root and ascending aorta diameters. However, in this study we used linear regression analysis to assess the dilation rate following the previously described instrumental variables approach which mitigates problems associated with random errors such as measurement variability [19]. There was a wide variety in the type and doses of drugs taken by the patients included in the present study. Also within patients, the type and dose of drug could change during follow-up. Therefore a more precise analysis per type and dose of statins was not feasible. Future studies may provide further insight into the differences between types of statins and their effects on the aortic root dilation rate.

## Conclusions

In conclusion, in patients with BAV, the use of statins is associated with smaller STJ and AAo diameters when corrected for confounding factors. However, the annual growth rate of the aortic root and ascending aorta was not influenced by the use of statins.
